# General contextual effects on neglected tropical disease risk in rural Kenya

**DOI:** 10.1371/journal.pntd.0007016

**Published:** 2018-12-21

**Authors:** William A. de Glanville, Lian F. Thomas, Elizabeth A. J. Cook, Barend M. de C. Bronsvoort, Nicola Wardrop, Claire N. Wamae, Samuel Kariuki, Eric M. Fèvre

**Affiliations:** 1 Centre for Immunity, Infection and Evolution, Institute for Immunology and Infection Research, School of Biological Sciences, University of Edinburgh, Edinburgh, United Kingdom; 2 International Livestock Research Institute, Nairobi, Kenya; 3 The Roslin Institute, Royal (Dick) School of Veterinary Studies, University of Edinburgh, Roslin, Midlothian, United Kingdom; 4 Department of Geography and Environment, University of Southampton, Highfield Campus, Southampton, United Kingdom; 5 School of Pharmacy and Health Sciences, United States International University, Nairobi, Kenya; 6 Centre for Microbiology Research, Kenya Medical Research Institute, Nairobi, Kenya; 7 Institute of Infection and Global Health, University of Liverpool, Leahurst Campus, Neston, United Kingdom; RTI International, UNITED REPUBLIC OF TANZANIA

## Abstract

The neglected tropical diseases (NTDs) are characterized by their tendency to cluster within groups of people, typically the poorest and most marginalized. Despite this, measures of clustering, such as within-group correlation or between-group heterogeneity, are rarely reported from community-based studies of NTD risk. We describe a general contextual analysis that uses multi-level models to partition and quantify variation in individual NTD risk at multiple grouping levels in rural Kenya. The importance of general contextual effects (GCE) in structuring variation in individual infection with *Schistosoma mansoni*, the soil-transmitted helminths, *Taenia* species, and *Entamoeba histolytica/dispar* was examined at the household-, sublocation- and constituency-levels using variance partition/intra-class correlation co-efficients and median odds ratios. These were compared with GCE for HIV, *Plasmodium falciparum* and *Mycobacterium tuberculosis*. The role of place of residence in shaping infection risk was further assessed using the spatial scan statistic. Individuals from the same household showed correlation in infection for all pathogens, and this was consistently highest for the gastrointestinal helminths. The lowest levels of household clustering were observed for *E*. *histolytica/dispar*, *P*. *falciparum* and *M*. *tuberculosis*. Substantial heterogeneity in individual infection risk was observed between sublocations for *S*. *mansoni* and *Taenia solium* cysticercosis and between constituencies for infection with *S*. *mansoni*, *Trichuris trichiura* and *Ascaris lumbricoides*. Large overlapping spatial clusters were detected for *S*. *mansoni*, *T*. *trichiura*, *A*. *lumbricoides*, *and Taenia* spp., which overlapped a large cluster of elevated HIV risk. Important place-based heterogeneities in infection risk exist in this community, and these GCEs are greater for the NTDs and HIV than for TB and malaria. Our findings suggest that broad-scale contextual drivers shape infectious disease risk in this population, but these effects operate at different grouping-levels for different pathogens. A general contextual analysis can provide a foundation for understanding the complex ecology of NTDs and contribute to the targeting of interventions.

## Author summary

Variation in infectious disease risk between groups of individuals represent health inequalities: reducing these inequalities, alongside reductions in infection prevalence, is a major focus for public health interventions. Despite this, it is rare that general contextual effects, or measures of within-group correlation or between-group heterogeneity, are reported as substantive outcomes from community-based studies of infectious disease risk, including for the NTDs. This reflects wider issues around a lack of social epidemiological perspectives, or consideration of the effects of contextual drivers, in communicable disease research, particularly in low-income settings. The aim of this study was to measure general contextual effects on human infection risk for a number of endemic helminth, protozoal, bacterial and viral pathogens in a rural farming community in western Kenya. Using this approach, we reveal clustering at a range of administrative and geographic levels and are able to show that the magnitude of clustering, and the hierarchical grouping level at which it occurs (from the household to administrative constituency), varies substantially between pathogens. Greater within-group correlation and between-group heterogeneity in infection risk was observed for the helminth NTDs and HIV than for *Entamoeba histolytica/dispar*, *Plasmodium falciparum* or *Mycobacterium tuberculosis*. Quantification of general contextual effects can inform the design of interventions that aim to reduce health inequalities within a population and can provide actionable targets for assessing the short- and long-term impact of interventions.

## Introduction

People living in rural areas in sub-Saharan Africa are often at high risk of infection with a range of pathogens [1–3]. The burden of preventable infectious disease in many of these communities can perpetuate poverty [4], reduce well-being [5,6], and contribute to high rates of mortality [7]. An individual’s risk of infection with any pathogen depends on a complex interplay of factors that relate to their exposure and susceptibility [8]. The individual-level characteristics that determine the likelihood of encountering a particular pathogen, and of infection following exposure, are often greatly influenced by the social, cultural, political, economic and/or environmental contextual conditions in which a person lives [9–11]. Since individuals living in the same geographic, administrative or institutional setting are generally exposed to the same contextual conditions (although not necessarily in the same way), adverse health outcomes commonly cluster within particular grouping levels. Hence, all else being equal, two people living in the same group will tend to be more similar in their health status than two people living in different groups [12]. Such clustering effects are often large for infectious diseases, and particularly so at the household-level for pathogens that are spread through poor sanitation, contaminated water, endophagic vectors, and unhygienic practices [13–18].

Clustering of infection within groups, and the contextual effects that drive it, such as marginalization, poverty and access to health services, is integral to the conceptualization of an infectious disease as ‘neglected’ [19]. However, it has been suggested that effects acting at the group-level are often forgotten in the epidemiological study of NTD infection risk [20,21], or indeed of infectious disease risk more broadly [22,23]. This apparent deficit in “contextual thinking” has occurred despite the widespread use of multi-level models, also called random effect or hierarchical models, in community-based studies of infectious disease risk in low income settings. One possible explanation for the absence of explicit contextual thinking is that the condition that necessitates the use of random effects in these multi-level models, namely the presence of within-group correlation in the outcome of interest, is almost never reported as an outcome of substantive interest in studies of NTD risk. Intra-group correlation (and the need for group-level random effects) is evidence that the grouping level chosen, be it household or geographic or administrative area, has a role in shaping variation in risk, and therefore points to the importance of group-level effects on individual infection.

A number of authors have described the value (and, it could be argued, the need [24]) of considering and reporting measures of general contextual effect, such as within-group correlation or between-group heterogeneity, from multi-level studies of disease risk [12,24–29]. Such effects are described as “general” as they refer only to influence of the cluster boundaries, rather than the specific contextual characteristics of the cluster [28]. Quantification of the extent and level at which infection risk varies between these clusters of individuals can contribute to the development of research questions that are explicitly contextual, and which therefore seek to better understand how the conditions in which people live impact upon their health [27,28]. Moreover, if health inequalities can be defined as differences in health status between groups of individuals [30], estimating general contextual effects (GCE), such as the median odds ratio or the intra-cluster correlation coefficient, can also provide a simple and standardized means with which to quantify and compare health inequalities within and between populations, and for different health outcomes [31]. Estimation of these group-level effects is straightforward to integrate into the multi-level analysis of community-based disease risk [32–35], and can provide fundamental information on the levels of variation that exist within a population.

Here, we describe a general contextual analysis that seeks to quantify the role of group-level effects in shaping variation in endemic NTD risk at a range of levels of aggregation in a rural farming community in Kenya. Since the NTDs commonly co-occur with HIV/AIDS, tuberculosis (TB) and malaria [36], we compare the GCE observed for NTDs with infection with pathogens causing these three diseases. In addition to describing the levels of variation in helminth, bacterial, protozoal and viral infection risk that exists within a single population, our aim is to use this analysis to demonstrate the value that can be added to the multi-level analysis of NTD risk through the quantification of GCE.

## Methods

Data were collected as part of the ‘People, Animals and their Zoonoses’ (PAZ) study [37]. This was a large cross-sectional survey of all eligible and consenting members of 416 randomly selected households in a single, mixed farming community in western Kenya. In total, 2113 people of all ages meeting the inclusion criteria (≥ 5 years and without conditions that may have made blood sampling harmful) were included and sampled between September 2010 and July 2012. Samples from participants were tested for current infection with a range of pathogens. A questionnaire was conducted with all recruited participants in their preferred language (Kiswahili, Dholuo, Kiluhya or English).

### Grouping levels

**Fig 1:**
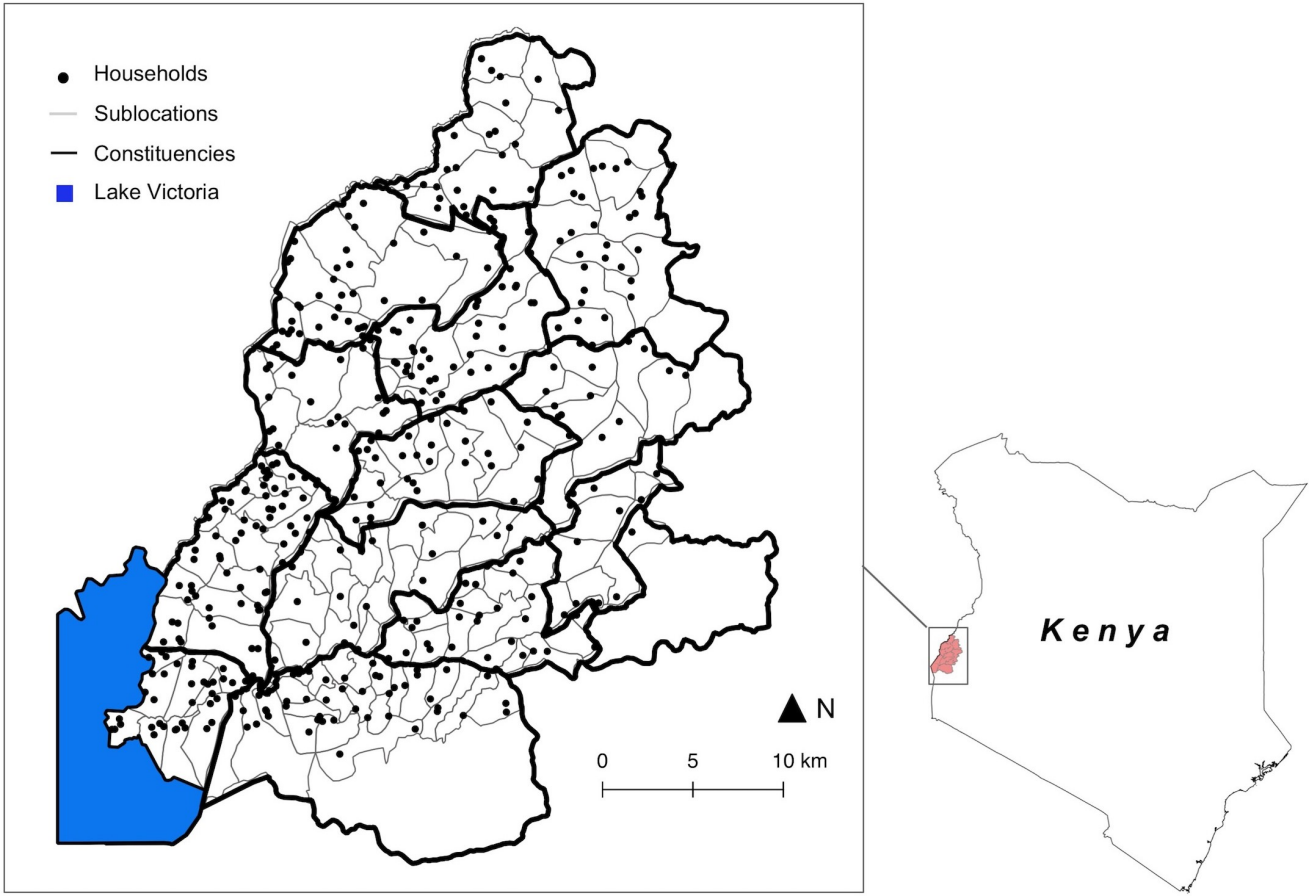
The geographic distribution of sampled households, sublocations and constituencies (Base layers from https://gadm.org/index.html).

Sampled individuals were nested within randomly selected households. These represented family groups living within a single compound, sharing meals and a common water source. The average reported household size was 7.6 people (range 1 to 30), from which our average sample size was 5.1 (range 1 to 21). Households were selected from within sublocations, the smallest administrative unit in Kenya. We sampled between 1 and 8 households in all 141 sublocations in the study area. The PAZ study focused on zoonotic disease risk, and the number of households selected per sublocation was proportional to the cattle population (see [37] for further details). Sublocations were nested within constituencies, the level at which government funding for development, and particularly for poverty alleviation, is allocated in Kenya. There were a total of 13 constituencies in the study area. On the basis of the 2009 census (OpenData, http://www.opendata.go.ke), sublocations in the study area had a median total population of 4,809 (range 1,187–33,352) and a median area of 10.8 km^2^ (range 0.96–64.6). Constituencies were made up of between 7 and 22 sublocations. The geographic distribution of sampled households, sublocations and constituencies is shown in Fig 1.

### Classifying outcomes

Several infectious agents, including a number that cause NTDs, were highly prevalent in the population under study. Here we focus on those with an individual-level prevalence after adjustment for the complex study design (see [37] for details) greater than 5%. These were *Ancylostoma duodenale* and/or *Necator americanus* (hereafter, hookworm) (36.3% (95% CI 32.8–39.9)); *Entamoeba histolytica/dispar* (30.1% (95% CI 27.5–32.8)); *Plasmodium falciparum* (29.4% (95% CI 26.8–32.0)); *Taenia* spp. (causing taeniasis) (19.7% (95% CI 16.7–22.7)); *Taenia solium* (causing cysticercosis) (5.8% (95% CI 4.4–7.2)); *Ascaris lumbricoides* (10.4% (95% CI 8.1–12.7)); *Trichuris trichiura* (10.0% (95% CI 8.2–11.7)); *Mycobacterium tuberculosis* (8.2% (95% CI 6.8–9.6)); *Schistosoma mansoni* (5.9% (95% CI 3.7–8.1)); and HIV (5.3% (95% CI 4.2–6.3)).

Individuals were classified as infected with *P*. *falciparum*, the only agent of malaria identified in the study area, if parasites were observed by light microscopy on thick or thin blood smears stained with Giemsa. Infection with the soil-transmitted helminths (hookworm, *A*. *lumbricoides*, *T*. *trichiura*) and *S*. *mansoni* was defined as the presence of at least one egg in a single faecal sample examined following preparation using the Kato-Katz (KK) [38] and formal ether concentration (FEC) techniques [39]. Infection with *E*. *histolytica/dispar* was defined as the presence of at least one cyst in a single faecal sample prepared using the FEC technique. *M*. *tuberculosis* infection was determined using a gamma-interferon assay (QuantiFERON-TB test, Cellestis) and HIV infection diagnosed using a rapid strip test (SD Bioline HIV 1/2 3.0, Standard Diagnostics). Infection with *Taenia* species (causing taeniasis, or the presence of an adult tapeworm in the gastrointestinal tract) was defined on the basis of a non-species specific copro-antigen ELISA [40], whilst cysticercosis due to *T*. *solium* (the presence of encysted larvae) was determined using a HP10-Ag ELISA on serum [41].

### Ethical approval

Ethical approval for this study was granted by the Kenya Medical Research Institute (KEMRI) Ethical Review Board (SCC1701). All participants or their guardians provided written informed consent. Individuals found to be infected with helminths or protozoa (including *P*. *falciparum*) were offered treatment free of charge by study clinical officers. Referral to local health facilities was provided where necessary.

### Model specification

The entire sample of 2113 people was used for the general contextual analysis. Missing-ness was present in all outcome measures and ranged from 0.05% (for *P*. *falciparum*) to 11.1% (for *M*. *tuberculosis*). Missing-ness was related to an absence of a particular sample type (blood or faeces), typically due to inadequate volumes collected or because of participant unwillingness to provide it.

Four-level logistic regression models were specified with infection as a binary outcome (infected/not infected) for each pathogen. Probability of infection was related to a set of predictors at the individual-level and random effects at the household-, sublocation- and constituency-levels. These models estimated the log odds of individual infection together with the variance at the intercept for the household (*σ*^2^_*H*_), sublocation (*σ*^2^_*SL*_) and constituency (*σ*^2^_*C*_) levels for an individual *i* living in household *j* in sublocation *k* in constituency *l*. The regression equation can be summarised as *logit*(*π*_*ijkl*_) = *β*_0_ + *βX* + *H*_0*jkl*_ + *SL*_0*kl*_ + *C*_0*l*_. Our primary motivation for this analysis was to quantify general (rather than specific) contextual effects operating at each of the three grouping levels. However, age, sex, education status and ethnicity were included as fixed effects, *X*, at the individual level in order to assess the impact of within-household composition on between-group variation. Models with and without fixed effects were estimated for each pathogen. A quadratic term was included for the continuous predictor age (recorded as 5 year intervals) based on the expectation of non-linear relationships with infection risk for several pathogens [37]. The continuous age variable was scaled to have a mean of zero and standard deviation of one.

Models were estimated for each pathogen in WinBUGS 1.4.3 (http://www.mrc-bsu.cam.ac.uk/software/bugs/) using weakly informative normal priors for all fixed and random effects. The standard deviation for each of the group-level random effects was defined using a wide uniform hyper-prior (i.e. Uniform(1,100)). Model convergence was confirmed by visual assessment of MCMC chains. Inference was based on 3 chains that were allowed to run for at least 70,000 iterations after a burn-in of at least 30,000 with a thinning interval of at least 10. We derived the median and 2.5^th^ and 97.5^th^ percentiles from posterior distributions of each parameter for point estimates and 95% credibility intervals, respectively. All data manipulation was performed in R statistical environment (R version 3.1.1, http://cran.r-project.org/) with logistic regression models estimated via the *R2WinBUGs* package [42]. Estimation was performed within a Bayesian framework based on MCMC to reduce bias in the estimates for random effect parameters [43], and for ease of estimation of the associated uncertainty for GCE.

### Quantifying general contextual effects

#### Variance partition coefficient

The variance partition co-efficient (VPC) was calculated from the outputs from each multi-level logistic regression model for each pathogen using the latent variable method [44,45]. This approach assumes that the propensity for individual infection is on a continuous scale and that only those people for which a threshold is exceeded can be considered to acquire infection. Whilst it has been suggested that such a justification is difficult to make for truly discrete outcomes [44], such as infection, interacting thresholds relating to exposure (for example, infectious dose) and susceptibility (such as immunity) could be envisaged. The unobserved latent variable (or probability of infection) is assumed to follow a logistic distribution, with variance equal to *π*^2^/3 (i.e. 3.29). Using this approach, the VPC at the household (_H_), sublocation (_SL_) and constituency (_C_) levels were [27]:

$${VPC}_{H}=\frac{\sigma^{2}{}_{C}+\sigma^{2}{}_{SL}+\sigma^{2}{}_{H}}{\sigma^{2}{}_{C}+\sigma^{2}{}_{SL}+\sigma^{2}{}_{H}+\pi^{2}/3}$$

$${VPC}_{SL}=\frac{\sigma^{2}{}_{C}+\sigma^{2}{}_{SL}}{\sigma^{2}{}_{C}+\sigma^{2}{}_{SL}+\sigma^{2}{}_{H}+\pi^{2}/3}$$

$${VPC}_{C}=\frac{\sigma^{2}{}_{C}}{\sigma^{2}{}_{C}+\sigma^{2}{}_{SL}+\sigma^{2}{}_{H}+\pi^{2}/3}$$

The VPC represents the correlation in the probability of infection between two individuals randomly selected from the same household (*VPC*_*H*_), sublocation (*VPC*_*SL*_) or constituency (*VPC*_*C*_). For the models described, the VPC can be considered to be equivalent to the intra-class co-efficient (ICC).

In order to further evaluate the importance of higher contextual levels in structuring variation in individual infection, we also calculated the proportion of variance (PTV) at the sublocation and household level as a fraction of total variation:

$${PTV}_{H}=\frac{\sigma^{2}{}_{H}}{\sigma^{2}{}_{C}+\sigma^{2}{}_{SL}+\sigma^{2}{}_{H}+\pi^{2}/3}$$

$${PTV}_{SL}=\frac{\sigma^{2}{}_{SL}}{\sigma^{2}{}_{C}+\sigma^{2}{}_{SL}+\sigma^{2}{}_{H}+\pi^{2}/3}$$

We do not directly estimate *PTV*_*C*_ since this is equivalent to *VPC*_*C*_.

#### Median odds ratio

The median odds ratio (MOR) provides a measure of heterogeneity in an individual-level outcome between groups. It represents the median value of the odds ratio comparing group-level residuals from randomly selected pairs of individuals living in a group at higher risk and those from a group at lower risk [27]. The MOR can be considered to represent the (median) difference in odds when moving between groups. It can be calculated as [26]:

$${MOR}_{H}\approx exp(0.95*\sqrt{\sigma^{2}{}_{C}+\sigma^{2}{}_{SL}+\sigma^{2}{}_{H}})$$

$${MOR}_{SL}\approx exp(0.95*\sqrt{\sigma^{2}{}_{C}+\sigma^{2}{}_{SL}})$$

$${MOR}_{C}\approx exp(0.95*\sqrt{\sigma^{2}{}_{C}})$$

Where there is little variation in individual risk between groups, the MOR will be close to one.

### Spatial clustering

Geographic effects not captured in the non-spatial multi-level logistic regression models were identified by testing the standardised sublocation level residual log odds for evidence of spatial clustering in high or low values using the spatial scan statistic [46]. The default maximum cluster size of 50% of the sample was chosen using a circular spatial window. The sublocation was used as the highest contextual level for the exploration of spatial clustering due to the small number of groups at the constituency level (n = 13). We used a normal model in SatScan version 9.4.4 (www.satscan.org). To account for differences in sample sizes, the number of individuals sampled in each sublocation were included as model weights [47]. Sublocation residuals for spatial analysis were drawn from a three-level logistic regression model (with random effects for household and sublocation only) with and without adjustment for within-household compositional effects.

## Results

### General characteristics

The variation in prevalence of each infectious agent across the range of variables included as fixed effects is shown in Table 1. Variation in prevalence of infection between self-reported members the different ethnic groups was particularly apparent, and most notably so for *A*. *lumbricoides*, *T*. *trichiura*, *Taenia* spp. (causing taeniasis) and HIV. Heterogeneity in the prevalence of infection with each of these pathogens, and with *S*. *mansoni* and *T*. *solium* (causing cysticercosis), was also evident between constituencies.

**Table 1:** Participant characteristics and percentage infected with each pathogen.

	*AL*	HW	*TT*	*SM*	*EH*	*TA*	*TS*	HIV	*MT*	*PF*
**All**	8.8%	35.5%	11.2%	7.8%	29.9%	19.9%	6.6%	5.9%	8.0%	29.7%
**Sex**										
Female	9.3%	33.0%	13.1%	6.3%	32.3%	18.6%	7.7%	7.9%	7.6%	28.1%
Male	8.2%	38.4%	8.9%	9.7%	27.1%	21.4%	5.4%	3.6%	8.4%	31.7%
**Education**										
None	7.6%	46.2%	13.0%	6.5%	25.9%	19.1%	9.5%	11.3%	9.1%	16.2%
Primary	9.3%	37.4%	11.7%	8.5%	31.6%	19.6%	6.2%	6.0%	7.3%	31.9%
Beyond primary	7.4%	24.5%	8.3%	6.4%	25.6%	20.8%	6.7%	3.1%	9.8%	28.4%
**Ethnicity**										
Luhya	10.7%	37.6%	9.3%	9.6%	30.7%	17.7%	5.3%	4.3%	7.8%	28.2%
Luo	13.2%	28.6%	23.2%	6.8%	27.6%	32.5%	7.8%	12.3%	10.8%	29.5%
Samia	1.6%	32.0%	10.2%	9.4%	28.8%	17.3%	7.5%	5.0%	5.4%	36.0%
Teso	2.0%	41.2%	0.7%	2.0%	31.4%	11.3%	8.6%	2.6%	6.6%	30.1%
**Age group**										
5 to 9	13.9%	24.8%	10.9%	5.8%	26.4%	18.0%	6.5%	0.9%	1.8%	51.6%
10 to 14	10.6%	34.3%	15.1%	9.6%	31.9%	20.1%	7.1%	2.3%	3.5%	49.1%
15 to 24	8.5%	35.5%	13.4%	9.3%	35.0%	19.9%	5.5%	1.8%	8.1%	23.0%
25 to 39	6.0%	40.6%	8.5%	8.2%	28.1%	19.2%	6.2%	13.2%	14.1%	14.2%
40+	4.7%	42.7%	8.1%	6.7%	28.6%	22.1%	7.5%	11.5%	12.9%	9.0%
**Constituency**										
Alego Usonga	11.8%	23.1%	24.9%	10.7%	22.4%	43.9%	17.5%	12.4%	10.5%	31.6%
Budalangi	10.4%	24.8%	24.3%	30.2%	20.8%	21.4%	3.1%	11.5%	12.5%	25.7%
Bumula	11.4%	48.5%	0.6%	2.4%	31.6%	10.3%	6.7%	1.7%	2.7%	26.8%
Butula	14.7%	47.3%	16.0%	2.7%	32.9%	24.3%	7.1%	5.8%	6.8%	32.7%
Funyula	2.4%	32.3%	9.9%	9.6%	29.9%	17.7%	6.6%	4.2%	5.6%	36.2%
Matayos	15.3%	50.8%	7.6%	1.7%	32.2%	26.7%	4.8%	6.3%	10.3%	35.7%
Matungu	16.5%	34.1%	3.3%	7.7%	33.0%	15.4%	2.1%	3.2%	10.6%	36.2%
Mumias West	14.5%	37.3%	14.5%	7.2%	21.4%	21.4%	2.3%	1.1%	9.2%	21.6%
Nambale	3.3%	36.3%	0.9%	2.4%	41.0%	6.4%	7.0%	0.5%	7.3%	25.0%
Teso North	0.9%	37.0%	0.0%	1.9%	37.0%	10.5%	17.6%	1.9%	5.9%	31.5%
Teso South	2.4%	44.6%	4.8%	2.4%	27.9%	12.2%	5.1%	3.9%	4.1%	23.5%
Ugenya	10.9%	20.7%	14.1%	1.1%	27.2%	21.1%	0.0%	16.0%	15.3%	22.3%
Ugunja	16.8%	26.9%	24.4%	10.1%	31.4%	34.5%	1.6%	10.3%	11.7%	33.3%
**Total tested**	**2013**	**2013**	**2013**	**2013**	**2057**	**1993**	**2092**	**2105**	**1879**	**2112**

**AL** = *A*. *lumbricoides*; **HW** = Hookworm; **TT** = *T*. *Trichiura*; **SM** = *S*. *mansoni*; **EH** = *E*. *histolytica/dispar;* **TA** = *Taenia* spp.; **TS** = *T*. *solium*; **HIV** = HIV; **MT** = *M*. *tuberculosis*; **PF** = *P*. *falciparum*

### Fixed effects

Co-efficients from the adjusted models (M2) for each pathogen are shown in Table 2 (STH and *S*. *mansoni*), Table 3 (*E*. *histolytica*/*dispar*, *Taenia* spp. and *T*. *solium*) and Table 4 (HIV, *P*. *falciparum*, *M*. *tuberculosis*). Male gender was associated with increased odds of hookworm and *S*. *mansoni* infection, with weaker evidence for taeniasis. Females had greater odds of *T*. *trichiura*, *E*. *histolytica/dispar*, and HIV infection and *T*. *solium* cysticercosis. There was no evidence of a relationship between sex and *A*. *lumbricoides* (Table 2), *M*. *tuberculosis*, or *P*. *falciparum* (Table 4) infection. Hookworm (Table 2), *M*. *tuberculosis* and HIV (Table 4) infection increased with age, with evidence in each case of a negative quadratic effect. Infection declined with age for *T*. *trichiura*, *A*. *lumbricoides* (Table 2) and *P*. *falciparum* (Table 4*)*. Having an education beyond primary school tended to reduce odds of infection for the majority of pathogens under study, although this was only significant in the case of hookworm (Table 2). There were strong relationships between ethnicity and infection for several pathogens, including substantially reduced odds among people of Samia and Teso ethnicity for *A*. *lumbricoides* compared to the Luhya baseline. Odds of *T*. *trichiura* infection were reduced among people of Teso ethnicity and elevated among people of Luo ethnicity when compared to the Luhya baseline. The odds of HIV infection was also higher among individuals of Luo ethnicity than the Luhya baseline.

**Table 2:** Posterior median estimates and 95% credibility intervals from null (M1) and adjusted (M2) models examining individual infection with hookworm, *Ascaris lumbricoides*, *Trichuris trichiura* and *Schistosoma mansoni*.

	Hookworm		*A*. *lumbricoides*		*T*. *trichiura*		*S*. *mansoni*	
	M1	M2	M1	M2	M1	M2	M1	M2
**Intercept**	-0.8 (-1.1, -0.4)	-0.3 (-0.9, 0.3)	-3.4 (-4.3, -2.6)	-3.5 (-4.6, -2.4)	-3.2 (-4.3, -2.2)	-2.8 (-4.0, -1.8)	-5 (-6.5, -3.8)	-5.8 (-7.8, -4.1)
**Age**	-	0.5 (0.4, 0.7)*	-	-0.9 (-1.2, -0.6)*	-	-0.5 (-0.8, -0.3)*	-	0.2 (-0.1, 0.6)
**Age x Age**	-	-0.2 (-0.3, -0.1)*	-	0.2 (-0.0, 0.5)	-	0.1 (-0.0, 0.3)	-	-0.3 (-0.6, -0.1)*
**Male gender**	-	0.5 (0.2, 0.7)*	-	-0.2 (-0.6, 0.2)	-	-0.4 (-0.8, -0.1)*	-	1.2 (0.7, 1.7)*
**Luo ethnicity**^**1**^	-	-0.1 (-0.7, 0.4)	-	0.4 (-0.3, 1.2)	-	1.1 (0.4, 1.8)*	-	0.0 (-1.4, 1.3)
**Samia ethnicity**^**1**^	-	-0.2 (-0.8, 0.4)	-	-1.9 (-3.4, -0.5)*	-	0.1 (-0.7, 0.9)	-	-0.8 (-1.8, 0.3)
**Teso ethnicity**^**1**^	-	0.1 (-0.4, 0.7)	-	-1.7 (-3.1, -0.4)*	-	-2.2 (-4.3, -0.6)*	-	-1.9 (-4.0, 0.0)
**Primary**^**2**^	-	-0.4 (-0.9, 0.0)	-	0.0 (-0.8, 0.8)	-	-0.3 (-0.9, 0.4)	-	0.5 (-0.5, 1.6)
**Beyond primary**^**2**^	-	-1.2 (-1.7, -0.6)*	-	-0.4 (-1.3, 0.6)	-	-0.7 (-1.4, 0.1)	-	-0.3 (-1.5, 0.8)
***σ***^**2**^_***H***_	1.4 (0.9, 2)	1.5 (1.0, 2.2)	2.7 (1.6, 4.5)	3.1 (1.9, 5.0)	0.6 (0.0, 1.3)	0.4 (0.0, 1.1)	3.8 (2.0, 7.1)	5.0 (2.7, 9.4)
***σ***^**2**^_***SL***_	0.3 (0.0, 0.7)	0.4 (0.1, 0.8)	0.1 (0.0, 0.8)	0.1 (0.0, 0.8)	1.0 (0.5, 1.9)	1.0 (0.5, 1.9)	2.2 (0.6, 5.1)	3.1 (1.0, 6.6)
***σ***^**2**^_***C***_	0.2 (0.0, 0.8)	0.2 (0.0, 1.0)	1.2 (0.3, 4.0)	0.3 (0.0, 1.7)	2.7 (0.9, 9.0)	1.2 (0.3, 4.7)	2.6 (0.8, 9.3)	3.3 (0.9, 11.5)
**VPC**_**H**_	37.0% (29.5, 45.7)	39.6% (31.7, 49.1)	56.1% (43.8, 69.6)	52.5% (40.5, 65.2)	57.0% (41.4, 76.6)	45.0% (30.5, 65.9)	73.3% (61.8, 84)	78.2% (68.1, 87.3)
**VPC**_**SL**_	10.2% (3.2, 20.2)	11.8% (4.3, 23.5)	18.7% (6.7, 41.8)	6.8% (0.5, 23.9)	49.4% (31.4, 72.7)	38.2% (23.0, 61.6)	41.4% (23.7, 63)	44.6% (25.4, 65.3)
**VPC**_**C**_	3.9% (0.4, 13.4)	4.2% (0.2, 16)	15.7% (5.0, 39.1)	4.2% (0.0, 20.7)	35.0% (15.3, 64.7)	19.9% (5.5, 50)	21.6% (7.7, 48)	22.4% (7.5, 48.8)
**PTV**_**HH**_	26.5% (18.7, 35.3)	27.4% (19.1, 36.9)	36.2% (23.1, 49.6)	44.5% (31, 57.2)	7.1% (0.5, 16.5)	6.0% (0.0, 16.9)	31.1% (17.1, 48.1)	32.9% (18.2, 50.6)
**PTV**_**SL**_	5.7% (0.1, 12.6)	6.8% (1.2, 14.1)	1.7% (0.0, 10.6)	1.2% (0.0, 10.2)	13.2% (5.5, 24.5)	17.1% (7.4, 29.2)	18.0% (4.8, 34.7)	20.3% (6.8, 37.7)
**MOR**_**H**_	3.7 (3.1, 4.9)	4.0 (3.2, 5.4)	7 (4.6, 13.6)	6.1 (4.1, 10.6)	7.3 (4.3, 22.6)	4.8 (3.1, 11.0)	17.4 (8.9, 52.2)	26.1 (12.4, 91.6)
**MOR**_**SL**_	2.0 (1.5, 2.8)	2.1 (1.6, 3.2)	3.1 (1.9, 7.2)	1.9 (1.2, 3.8)	6.3 (3.6, 20.6)	4.2 (2.8, 10.0)	8.5 (4.3, 28.4)	11.5 (5.2, 43.7)
**MOR**_**C**_	1.5 (1.1, 2.3)	1.6 (1.1, 2.6)	2.8 (1.7, 6.7)	1.7 (1.1, 3.5)	4.7 (2.5, 17.3)	2.8 (1.7, 7.9)	4.7 (2.3, 18.3)	5.7 (2.5, 25.0)
**DIC**^**3**^	2523.4	2442.9	1121.3	1079.4	1308.6	1296.8	835.9	790.5

* 95% credibility intervals do not include 0

^1^ Luhya ethnicity baseline

^2^ No education baseline

^3^ Deviance information criteria (DIC) is an estimate of predictive error: the lower the better.

**Table 3:** Posterior median estimates and 95% credibility intervals from null (M1) and adjusted (M2) models examining individual infection with *Entamoeba histolytica/dispar*, taeniasis due to infection with *Taenia solium* or *T*. *saginata* and cysticercosis due to *T*. *solium*.

	*E*. *histolytica/dispar*		*Taenia* spp.		*T*. *solium*	
	M1	M2	M1	M2	M1	M2
**Intercept**	-1.0 (-1.2, -0.8)	-1.0 (-1.5, -0.5)	-2.1 (-2.7, -1.6)	-2.2 (-3.0, -1.4)	-4.7 (-5.7, -3.9)	-4.6 (-6.0, -3.4)
**Age**	-	0.0 (-0.1, 0.2)	-	0.1 (-0.1, 0.3)	-	-0.1 (-0.4, 0.2)
**Age x Age**	-	-0.1 (-0.2, 0.1)	-	0.0 (-0.2, 0.1)	-	0.2 (-0.1, 0.4)
**Male gender**	-	-0.3 (-0.5, -0.1)*	-	0.3 (-0.0, 0.6)	-	-0.7 (-1.1, -0.2)*
**Luo ethnicity**^**1**^	-	-0.2 (-0.5, 0.2)	-	0.1 (-0.6, 0.8)	-	0.1 (-0.9, 1.1)
**Samia ethnicity**^**1**^	-	-0.1 (-0.6, 0.4)	-	-0.4 (-1.1, 0.4)	-	0.4 (-0.9, 1.7)
**Teso ethnicity**^**1**^	-	0.0 (-0.4, 0.5)	-	-0.8 (-1.7, 0.0)	-	0.7 (-0.3, 1.7)
**Primary**^**2**^	-	0.3 (-0.1, 0.7)	-	0.0 (-0.5, 0.6)	-	-0.5 (-1.3, 0.3)
**Beyond primary**^**2**^	-	0.0 (-0.5, 0.5)	-	0.1 (-0.5, 0.8)	-	-0.2 (-1.1, 0.7)
***σ***^**2**^_***H***_	0.7 (0.4, 1.1)	0.7 (0.4, 1.1)	1.6 (1.0, 2.7)	1.8 (1.1, 2.9)	1.1 (0.3, 2.5)	1.4 (0.4, 3.0)
***σ***^**2**^_***SL***_	0.0 (0.0, 0.2)	0.0 (0.0, 0.2)	1.6 (0.8, 2.9)	1.6 (0.8, 2.8)	4.3 (2.4, 7.9)	4.7 (2.6, 9.0)
***σ***^**2**^_***C***_	0.0 (0.0, 0.2)	0.0 (0.0, 0.3)	0.5 (0.1, 2.0)	0.3 (0.0, 1.5)	0.3 (0.0, 2.3)	0.3 (0.0, 2.5)
**VPC**_**H**_	19.4% (13.2, 26.8)	20.1% (13.5, 27.7)	54.0% (44.6, 64.1)	54.0% (44.6, 63.6)	64.2% (51.9, 76)	67.0% (55.2, 77.9)
**VPC**_**SL**_	2.5% (0.3, 8.1)	2.5% (0.2, 8.4)	30.5% (18.8, 44.8)	27.9% (16.1, 41.8)	51.7% (37.3, 66.3)	52.7% (37.5, 68.3)
**VPC**_**C**_	1.1% (0.0, 5.8)	1.1% (0.0, 5.9)	7.2% (0.8, 23.5)	4.2% (0.0, 18.7)	2.8% (0.0, 21)	2.6% (0.0, 20.8)
**PTV**_**HH**_	16.5% (10.2, 23.8)	17.2% (10.3, 24.6)	23.0% (14.1, 34)	25.7% (16.2, 37.2)	11.8% (4.0, 23.4)	13.8% (4.8, 26.4)
**PTV**_**SL**_	0.9% (0.0, 5.3)	0.9% (0.0, 5.8)	21.9% (11.0, 35.5)	22.4% (10.8, 35.3)	47.0% (31.0, 62.6)	48.1% (31.8, 64.3)
**MOR**_**H**_	2.3 (2.0, 2.8)	2.4 (2.0, 2.9)	6.5 (4.7, 10.0)	6.5 (4.7, 9.8)	10.1 (6.0, 21.4)	11.7 (6.8, 25.5)
**MOR**_**SL**_	1.4 (1.1, 1.7)	1.4 (1.1, 1.8)	4.1 (2.8, 6.6)	3.8 (2.6, 6.0)	7.9 (4.8, 16.9)	8.8 (5.1, 20.1)
**MOR**_**C**_	1.2 (1.0, 1.6)	1.2 (1.0, 1.6)	2.0 (1.3, 3.9)	1.7 (1.0, 3.3)	1.6 (1.0, 4.2)	1.6 (1.0, 4.4)
**DIC**^**3**^	2701.3	2704.9	1731.5	1731.7	838.7	822.3

* 95% credibility intervals do not include 0

^1^ Luhya ethnicity baseline

^2^ No education baseline

^3^ Deviance information criteria (DIC) is an estimate of predictive error: the lower the better.

**Table 4:** Posterior median estimates and 95% credibility intervals from null (M1) and adjusted (M2) models examining individual infection with HIV, Plasmodium falciparum, and *Mycobacterium tuberculosis*.

	**HIV**		***P*. *falciparum***		***M*. *tuberculosis***	
	**M1**	**M2**	**M1**	**M2**	**M1**	**M2**
**Intercept**	-3.3 (-4.1, -2.6)	-2.8 (-3.8, -1.9)	-1 (-1.2, -0.8)	-1.8 (-2.4, -1.2)	-2.6 (-3.0, -2.3)	-2.9 (-3.8, -2.2)
**Age**	-	1.7 (1.3, 2.1)*	-	-1.5 (-1.7, -1.3)*	-	1.1 (0.8, 1.4)*
**Age x Age**	-	-0.9 (-1.2, -0.7)*	-	0.4 (0.3, 0.6)*	-	-0.4 (-0.6, -0.3)*
**Male gender**	-	-0.5 (-1.0, -0.1)*	-	0.1 (-0.1, 0.3)	-	0.3 (-0.1, 0.6)
**Luo ethnicity**^**1**^	-	1.1 (0.4, 1.8)*	-	0.2 (-0.2, 0.6)	-	0.3 (-0.3, 0.8)
**Samia ethnicity**^**1**^	-	0.6 (-0.3, 1.6)	-	0.2 (-0.4, 0.7)	-	-0.2 (-1.1, 0.7)
**Teso ethnicity**^**1**^	-	-0.1 (-1.2, 0.9)	-	0.0 (-0.4, 0.5)	-	0.1 (-0.6, 0.9)
**Primary**^**2**^	-	-0.3 (-1.0, 0.4)	-	0.1 (-0.4, 0.7)	-	0.2 (-0.4, 0.9)
**Beyond primary**^**2**^	-	-0.7 (-1.6, 0.1)	-	-0.2 (-0.7, 0.4)	-	0.6 (-0.2, 1.3)
***σ***^**2**^_***H***_	0.3 (0.0, 1.3)	0.4 (0.0, 1.7)	0.3 (0.1, 0.6)	0.4 (0.1, 0.8)	0.2 (0.0, 0.9)	0.4 (0.0, 1.2)
***σ***^**2**^_***SL***_	0.3 (0.0, 0.8)	0.3 (0.0, 1.0)	0.2 (0.0, 0.3)	0.2 (0.0, 0.4)	0.0 (0.0, 0.3)	0.1 (0.0, 0.4)
***σ***^**2**^_***C***_	1.0 (0.3, 3.5)	0.6 (0.1, 2.3)	0.0 (0.0, 0.2)	0.0 (0.0, 0.3)	0.1 (0.0, 0.6)	0.2 (0.0, 0.9)
**VPC**_**H**_	34.7% (19, 56.9)	30.6% (14.0, 52.1)	12.7% (7.1, 19.7)	17.6% (10.3, 26.2)	13.5% (3.6, 27.7)	18.7% (5.7, 35.4)
**VPC**_**SL**_	26.8% (11.8, 51.7)	20.2% (6.6, 43.2)	4.9% (1.2, 10.3)	6.4% (1.5, 13.5)	5.9% (0.7, 17.6)	6.8% (0.8, 21.5)
**VPC**_**C**_	20.5% (7.7, 47.6)	12.3% (2.1, 35.9)	0.5% (0.0, 3.9)	1.0% (0.0, 6.7)	3.6% (0.1, 14.7)	4.1% (0.0, 18.8)
**PTV**_**HH**_	6.2% (0.1, 22.5)	8.8% (0.1, 27.9)	7.6% (2.1, 14.2)	10.8% (3.9, 19.1)	6.4% (0.1, 19.6)	10.8% (0.3, 24.8)
**PTV**_**SL**_	4.9% (0.1, 16)	6.4% (0.1, 19.8)	4.0% (0.6, 8.8)	4.9% (0.6, 10.8)	1.2% (0.0, 8.8)	1.5% (0.0, 9.9)
**MOR**_**H**_	3.5 (2.3, 7.3)	3.1 (2.0, 6)	1.9 (1.6, 2.3)	2.2 (1.8, 2.8)	2 (1.4, 2.9)	2.3 (1.5, 3.6)
**MOR**_**SL**_	3.0 (2.0, 6.5)	2.5 (1.6, 4.9)	1.5 (1.2, 1.8)	1.6 (1.3, 2.1)	1.6 (1.2, 2.3)	1.6 (1.2, 2.6)
**MOR**_**C**_	2.6 (1.7, 6)	2.1 (1.3, 4.3)	1.1 (1.0, 1.5)	1.2 (1.0, 1.6)	1.4 (1.0, 2.1)	1.5 (1.0, 2.5)
**DIC**^**3**^	1101.5	1036.2	1275.5	1276.8	2883	2545.8

* 95% credibility intervals do not include 0

^1^ Luhya ethnicity baseline

^2^ No education baseline

^3^ Deviance information criteria (DIC) is an estimate of predictive error: the lower the better

### General contextual effects

**Fig 2:**
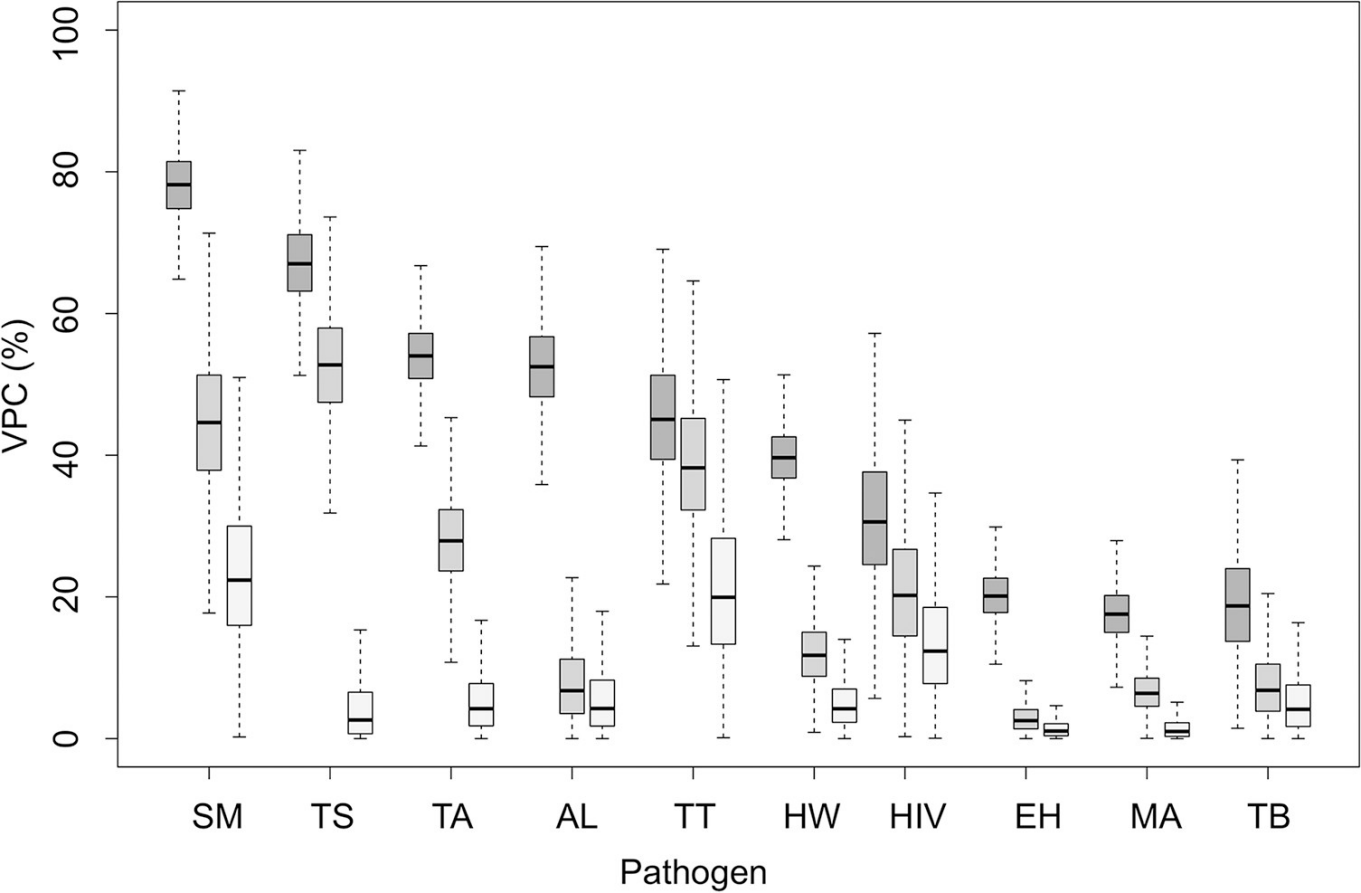
Posterior distributions of variance partition coefficients (VPC) for each pathogen at the household (dark grey), sublocation (grey) and constituency levels (light grey) without control for individual-level predictors (**SM** = *S*. *mansoni*; **TS** = *T*. *solium*; **TA** = *Taenia* spp.; **AL** = *A*. *lumbricoides*; **TT** = *T*. *Trichiura*; **HW** = Hookworm; **HIV** = HIV; **EH** = *E*. *histolytica/dispar;* **MA** = *P*. *falciparum;* **TB** = *M*. *tuberculosis*).

The posterior distribution of household-, sublocation- and constituency-level variance, VPCs, MORs and PTVs for the gastrointestinal nematodes and *S*. *mansoni* are shown in Table 2, in Table 3 for *E*. *histolytica/dispar* and *Taenia* species, and in Table 4 for HIV, *P*. *falciparum* and *M*. *tuberculosis*. Some degree of clustering at the household-level was apparent for all pathogens. This was consistently highest for the helminth parasites (Fig 2), for which there was substantial heterogeneity in risk of infection between individuals in different households, as evidenced by MORs which exceeded 3.5 for each helminth infection in both the null and adjusted models (Table 2 and Table 3). To put these effects into context, we would expect that were an individual to permanently move from one household to another with higher risk anywhere in the study area, their odds of infection with the helminth parasites under study would change by at least 3.5 times. This household clustering effect was particularly large for *S*. *mansoni* (Table 2) and *T*. *solium* cysticercosis (Table 3). The partitioning of group-level variation was generally largest at the household-level, although the greatest proportion of individual variation was partitioned at the constituency level (VPC_c_) in null models for *T*. *trichiura* (Table 2) and HIV (Table 4), and the sublocation-level for *T*. *solium* cysticercosis (PTV_SL_) (Table 3). Using MORs, these higher-level contextual effects could be interpreted as an almost five- and three-fold change in the odds of infection for an individual that permanently moves to a higher risk constituency for *T*. *trichiura* and HIV, respectively. Similarly, the median odds of an individual permanently moving to a higher risk sublocation could be expected to increase by around eight times for *T*. *solium* cysticercosis. Control for individual-level fixed effects resulted in declines in within-constituency correlation (VPC_C_) and between-constituency heterogeneity (MOR_C_) for infection with several of the pathogens under study, most notably for *A*. *lumbricoides* and *T*. *trichiura* (Table 2) and HIV (Table 4).

### Spatial clustering

**Fig 3:**
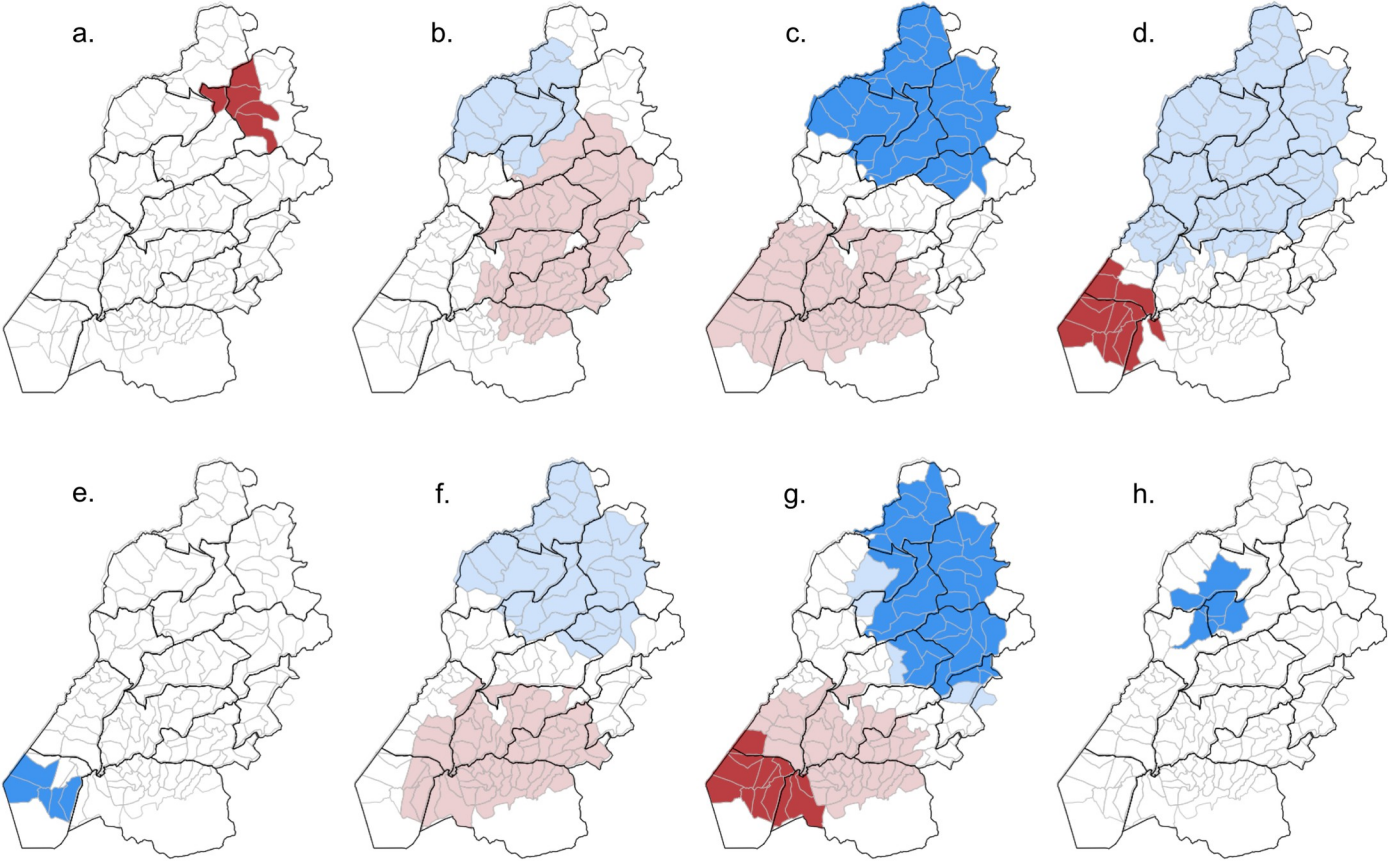
Clusters of significantly elevated (red) and reduced (blue) sublocation level standardised residual log odds of infection for: a. Hookworm; b. *A*. *lumbricoides*; c. *T*. *trichiura;* d. *S*. *mansoni;* e. *E*. *histolytica/dispar*; f. *Taenia* spp.; g. HIV; h. *P*. *falciparum*. Light and dark shades of red and blue represent significant clusters from the null and adjusted logistic regression models, respectively.

The spatial distribution of sublocations with evidence for clustering in high or low values of residual log odds of infection is shown in Fig 3. Large spatial clusters of both high and low values were observed from null models for *T*. *trichiura*, *S*. *mansoni*, *A*. *lumbricoides*, and *Taenia* spp.. There was substantial overlap in clusters for all of these pathogens and a large cluster of sublocations with elevated risk of individual HIV infection. We found no evidence of spatial structuring in the sublocation-level residual log odds of infection with *M*. *tuberculosis* or *T*. *solium* and relatively small clusters for *P*. *falciparum*, hookworm and *E*. *histolytica/dispar* (Fig 3). The spatial extent of the clusters of both high and low sublocation residual log odds was reduced when controlling for individual-level fixed effects in the case of HIV. Adjustment for these fixed effects resulted in a loss of significance in spatial clusters of both high and low values from the model for *A*. *lumbricoides*, and of high values for *T*. *trichiura*. Only the spatial cluster of positive sublocation residual log odds remained significant in the case of *S*. *mansoni* (Fig 3).

## Discussion

In this general contextual analysis, we demonstrate the value of summarizing variation in individual infectious disease risk at one or more biologically relevant grouping levels using the outputs from multi-level regression. Deriving statistics such as the MOR and VPC (or ICC) as part of an exploratory analysis of infectious disease risk is straightforward, and can contribute important information about the heterogeneity that underlies population-level averages, such as prevalence [26–28,33]. Using this approach, we show that variation in individual infection risk is partitioned at the household, sublocation and constituency-levels for a range of NTDs in a rural population in Kenya. These findings point to the importance of social and/or environmental contextual conditions in shaping infection at each of these levels, and which may provide actionable targets for public health interventions seeking to reduce both the prevalence of infection and the health inequalities observed.

An important limitation that should be recognised when interpreting these findings, and particularly when making comparisons between pathogens, is the lack of precision in many of our estimates of GCE, particularly at higher contextual levels. Hence, whilst estimates of VPC and MOR at the constituency-level were substantially different between, for example, hookworm and *S*. *mansoni* infection, the 95% credibility intervals overlap. This is a limitation of the sample available, both in terms of number of individuals and number of individual groups at the higher contextual levels. The magnitude of the MOR or VPC provides useful information on the importance of a particular level in structuring risk [28], and for the example of hookworm and *S*. *mansoni*, strongly suggests contextual drivers operating at the constituency level are more important for the latter than the former. However, when interpreting differences between pathogens at these higher contextual levels, or between different contextual levels for the same pathogen, it should be noted that the statistical support for many of the differences we observed was often limited.

A general contextual analysis can provide a tool for exploring the levels at which pathogen transmission occurs within a population [16]. For example, we show that the majority of variation in individual hookworm infection was partitioned at the household level, with comparatively smaller amounts at sublocation and constituency levels. This suggests clustering at higher contextual levels is less important for this parasite in this population than for the other STHs. Individual infection with *A*. *lumbricoides*, for example, was partitioned at both the household- and constituency-levels, and therefore household clusters of infection can also be considered to cluster by constituency. Household clustering was less important for *T*. *trichiura*, but there was substantial variation in infection between constituencies, and to a lesser extent between sublocations within constituencies. Understanding these patterns of partitioning in infection risk may assist in the design of interventions that seek to reduce both the prevalence and health inequalities observed. For pathogens with limited evidence for higher level GCE, such as hookworm or *E*. *histolytica/dispar*, it is likely that households in all parts of the study area would need to be targeted. Interventions in high risk constituencies are likely to be more cost effective for *T*. *trichiura*, *A*. *lumbricoides* and *S*. *mansoni*, potentially including a focus in high risk sublocations for the latter two pathogens. The general contextual analysis approach described here could be particularly valuable in monitoring the effectiveness of an intervention, such as mass drug administration. For example, a decline in population-level prevalence but persistence of, or increase in, general contextual effects at particular grouping-levels would point to ongoing or new health inequalities. Moreover, such a finding would suggest the presence of hotspots of transmission that may impact elimination [48]. Wider usage of general contextual analysis in the study of NTD risk could therefore contribute to the post-2020 NTD roadmap that sees a transition from monitoring programme coverage to measuring impact [49].

Clustering in *T*. *solium* cysticercosis and *Taenia* spp. taeniasis was observed at both the household and sublocation levels. This was particularly large at the sublocation level for *T*. *solium* cysticercosis, but not between constituencies. Hence, while spatially heterogeneous factors appear to influence cysticercosis risk, these effects are likely to operate at small spatial scales (i.e. at the sublocation-level). Cases of human cysticercosis commonly cluster around human tapeworm carriers [50], and Okello *et al* [51] reported hyper-endemic hotspots for *T*. *solium* infection in Lao PDR. The importance of non-spatially-structured sublocation effects in our own study area could therefore be hypothesised to reflect small-scale differences in pork consumption practices, or the existence of slaughterhouses in particular sublocations with inadequate meat inspection practices. Sublocation-level residuals for taeniasis showed substantial spatial structuring on the basis of the spatial scan statistic, and the lack of a similar finding for cysticercosis may point to a preponderance of the beef tapeworm, *T*. *saginata* (which does not cause human cysticercosis) over *T*. *solium* in the study area.

The nesting of variation in individual HIV infection at the constituency level supports the growing recognition that HIV epidemiology can be characterized as a number of diverse epidemics, often with substantial variation in prevalence even at small spatial scales [52,53]. In this part of western Kenya, individual risk of HIV infection was most concentrated in constituencies in the south-western part of the study area. Further work is needed to explore the important clustering observed, including the compositional effect of ethnicity; the Luo community who, as a group, have been previously been described to be heavily burdened by HIV [54], reside primarily in the southern part of the study area [37]. *Schistosoma haematobium*, which we did not test for but which is known to be an important co-factor for HIV infection in sub Saharan Africa [55], is also likely to be common in the swampy area around Lake Victoria [56], and may also contribute to the clustering observed. There were substantial overlaps in the spatial distribution of HIV infection risk and that for several NTDs, most notably *S*. *mansoni*, *A*. *lumbricoides* and *T*. *trichiura*. This supports earlier analysis of the same data that showed overlapping spatial clustering in household-level infection with these pathogens [37]. The observed co-distribution of these pathogens may point to the existence of shared environmental, cultural, behavioural or social conditions leading to poly-parasitism [19]. Alternatively, it may suggest immunological interactions between HIV and these helminth parasites that influence transmission dynamics, a hypothesis supported by a growing number of field and laboratory based studies [57].

Interestingly, between-group levels of variation were considerably lower for *P*. *falciparum* and *M*. *tuberculosis* than for any of the NTDs, with the exception of infection with *E*. *histolytica/dispar*. Previous studies on *M*. *tuberculosis* have suggested that the majority (>80%) of transmission events for the pathogen occurs in the public (or community) rather than domestic domain [58–60]. The comparatively small levels of individual variation partitioned at the household-level (particularly compared to the helminth pathogens under study) provides further support for these findings. Moreover, in the absence of higher level GCEs, we show there is little variation in community-level transmission between different parts of the study area for *M*. *tuberculosis*. Although we found evidence for a small cluster of sublocations with reduced risk of *P*. *falciparum* infection, the absence of higher-level contextual effects (at the sublocation- and constituency-level) for this pathogen suggests geographic or administrative place of residence does not have a major influence on infection risk. This is supported by a recent study from neighbouring Eastern Uganda which, using highly sensitive molecular-based diagnostic tests, demonstrated that the vast majority of community residents, regardless of age, demography and geographic location, were infected with malaria parasites [61].

We have explored only a limited set of fixed effects at the individual level in this analysis, and no specific contextual effects (i.e. predictors operating at group-level). Having demonstrated the importance of these grouping-levels in structuring infectious disease risk, the next analytical step would be to integrate specific contextual effects, including household, sublocation and constituency-level indicators of social or environmental conditions that may explain the variation observed. The inclusion of individual-level predictors resulted in substantial decreases in the variation at higher contextual levels for pathogens such as *A*. *lumbricoides*, *T*. *trichiura* and HIV. There were large, overlapping spatial clusters for each of these pathogens, the size of which was reduced or made to be non-significant following the inclusion of individual level predictors. All of these pathogens had strong relationships with ethnicity, which is known to be highly spatially structured in the study area [37]. Disentangling the importance of individual level cultural and behavioural practices and local social and environmental conditions would therefore help to better understand the general contextual effects observed.

### Conclusion

Quantification of general contextual effects provides a means to evaluate the importance of social and environmental conditions in structuring infectious disease risk within a population. Such an approach encourages the explicit consideration of group-level, contextual effects on individual health and can form the basis for subsequent analyses that seek to explain the variation observed. Using a general contextual analysis, we have demonstrated the existence of important place-based contextual effects for a range of pathogens in a rural farming community in Kenya and show that these are particularly large for the NTDs and HIV. This study provides evidence for important variation in infectious disease risk in this underprivileged population that point to the existence of health inequalities at a range of grouping-levels.

## Supporting information

S1 Checklist Strobe checklist. (DOC)
